# NUCKS overexpression in breast cancer

**DOI:** 10.1186/1475-2867-9-19

**Published:** 2009-08-10

**Authors:** Yiannis Drosos, Mirsini Kouloukoussa, Anne Carine Østvold, Kirsten Grundt, Nikos Goutas, Dimitrios Vlachodimitropoulos, Sophia Havaki, Panagoula Kollia, Christos Kittas, Evangelos Marinos, Vassiliki Aleporou-Marinou

**Affiliations:** 1 Department of Genetics and Biotechnology, Faculty of Biology, University of Athens, Panepistimioupoli, 15701 Ilissia, Greece; 2 Laboratory of Histology and Embryology, Medical School, University of Athens, 75 Mikras Asias Str., 11527 Goudi, Greece; 3 Department of Biochemistry, Institute of Basic Medical Sciences, University of Oslo, PO Box 1112, Blindern, 0317 Oslo, Norway; 4 Laboratory of Forensic Medicine and Toxicology, Medical School, University of Athens, 75 Mikras Asias Str., 11527 Goudi, Greece

## Abstract

**Background:**

NUCKS (Nuclear, Casein Kinase and Cyclin-dependent Kinase Substrate) is a nuclear, DNA-binding and highly phosphorylated protein. A number of reports show that *NUCKS *is highly expressed on the level of mRNA in several human cancers, including breast cancer. In this work, NUCKS expression on both RNA and protein levels was studied in breast tissue biopsies consisted of invasive carcinomas, intraductal proliferative lesions, benign epithelial proliferations and fibroadenomas, as well as in primary cultures derived from the above biopsies. Specifically, in order to evaluate the level of NUCKS protein in correlation with the histopathological features of breast disease, immunohistochemistry was employed on paraffin sections of breast biopsies of the above types. In addition, NUCKS expression was studied by means of Reverse Transcription PCR (RT-PCR), real-time PCR (qRT-PCR) and Western immunoblot analyses in the primary cell cultures developed from the same biopsies.

**Results:**

The immunohistochemical Results showed intense NUCKS staining mostly in grade I and II breast carcinomas compared to normal tissues. Furthermore, NUCKS was moderate expressed in benign epithelial proliferations, such as adenosis and sclerosing adenosis, and highly expressed in intraductal lesions, specifically in ductal carcinomas *in situ *(DCIS). It is worth noting that all the fibroadenoma tissues examined were negative for NUCKS staining. RT-PCR and qRT-PCR showed an increase of *NUCKS *expression in cells derived from primary cultures of proliferative lesions and cancerous tissues compared to the ones derived from normal breast tissues and fibroadenomas. This increase was also confirmed by Western immunoblot analysis. Although NUCKS is a cell cycle related protein, its expression does not correlate with Ki67 expression, neither in tissue sections nor in primary cell cultures.

**Conclusion:**

The results show overexpression of the NUCKS protein in a number of non malignant breast lesions and cancerous tissues. In particular, the NUCKS overexpression in ADH and DCIS indicates a significant role of this protein in neoplastic progression.

## Background

NUCKS (Nuclear, Casein Kinase and Cyclin-dependent kinase Substrate) is a nuclear, DNA-binding and highly phosphorylated protein. It has been shown that NUCKS is a substrate for CK2, Cdk1 and DNA-activated Kinase *in vitro *and *in vivo *[1-4]. Proteomic studies have revealed 25 phosphorylation sites and multiple sites of acetylation, methylation and formylation, suggesting that NUCKS has the highest ratio of modified to unmodified residues of any protein so far described [5]. The *NUCKS *gene is located on chromosome 1q32.1, lacks a TATA box but contains two Inr elements, two GC boxes and one consensus binding site for E2F1. Although the level varies, the *NUCKS *gene is ubiquitously expressed in all mammalian tissues and has many features of being a housekeeping gene [6]. NUCKS has been characterized as a cell cycle related protein that is synthesized in the M/G1 phase [7]. Even though it appears to be important for cell cycle progression, its exact role has not been yet clearly understood.

A number of reports show that *NUCKS *is highly expressed in several human cancers (ovarian, lung, bone marrow, brain) including breast cancer [8-10]. *NUCKS *gene belongs to a module of co-expressed genes located on chromosomal region 1q32.1, which is found to be amplified in breast cancer [10], as well as in other cancers [11,12]. Evidence that NUCKS is overexpressed in breast cancer with a possible role in regulating transcriptional regulation, makes it an excellent candidate molecule for study [8]. Moreover, it has been shown that *NUCKS *is overexpressed in post-selection human mammary epithelial cells (HMEC), an in vitro model of highly dysregulated epithelial cells with gene expression patterns highly similar to that of ductal carcinoma in situ (DCIS) and invasive breast cancer [13]. Furthermore, translational modifications of NUCKS revealed acetylation and methylation sites specific for breast cancer, resembling the data from core histones [5]. The above mentioned experimental data suggest that there is a relationship between *NUCKS *overexpression and breast cancer. However, no comprehensive study has been published up to day concerning the correlation of NUCKS expression with the histopathological type (i.e. DCIS, IDC, etc) and the tumor grading of breast cancer. In addition, all studies published so far, refer to the expression of mRNA, and there are no reports on the level of the NUCKS protein in different types of breast lesions.

In the present study, NUCKS expression has therefore been investigated in benign lesions, intraductal lesions and invasive carcinomas by means of immunohistochemistry, and in primary cell cultures of breast biopsies by measuring the levels of NUCKS mRNA and protein. Primary cell cultures have been developed from tissue fragments isolated directly from the tumor site, according to explant technique reflecting most of the aspects of tumor profile compared to established cell lines [14]. The results, taken altogether, show that NUCKS is overexpressed in the majority of invasive carcinomas and intraductal proliferations, mainly in DCIS.

## Results

### NUCKS immunolocalization

The distribution of breast tissue samples according to the histopathological type and grade is presented in Additional file 1.

**Figure 1:**
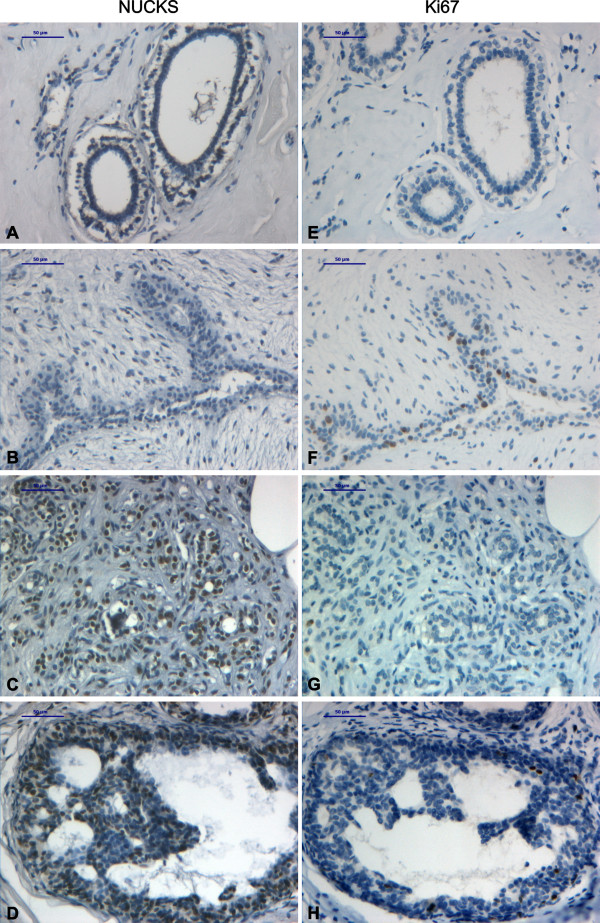
**Immunohistochemical staining of non malignant breast tissue biopsies with anti-NUCKS and anti-ki67 antibody**. Tissue sections stained with anti-NUCKS (A-D) and corresponding sections stained with anti-ki67 (E-H) antibody. (A), (E), Normal breast tissue exhibiting negative staining for both NUCKS and Ki67. (B), (F) Fibroadenoma exhibiting negative staining for NUCKS and low staining for Ki67. (C), (G) Sclerosing adenosis exhibiting strong staining for NUCKS and negative staining for Ki67. (D), (H) Strong immunoreaction for NUCKS in ADH and low for Ki67. Scale bar, 50 μm.

**Figure 2:**
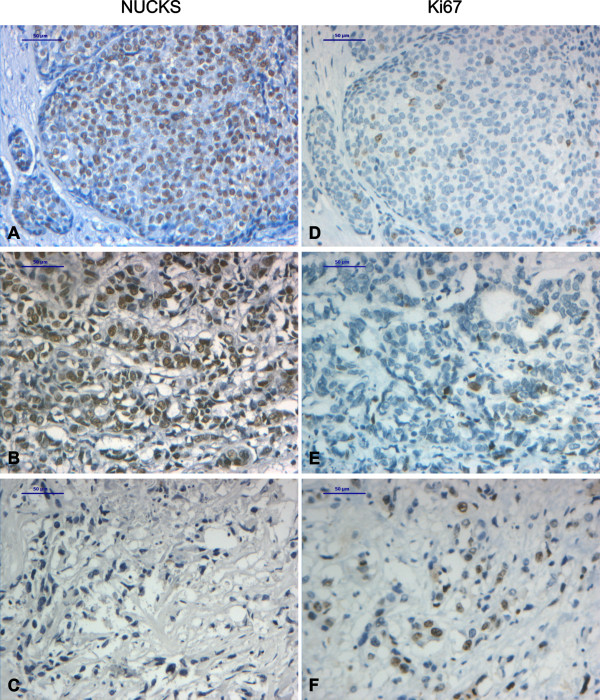
**Immunohistochemical staining of DCIS and IDC with anti-NUCKS and anti-ki67 antibody**. Tissue sections stained with anti-NUCKS (A-C) and corresponding sections stained with anti-ki67 (D-F) antibody. (A), (D), Strong immunoreaction for NUCKS and low for Ki67 in DCIS. (B), (E) Strong immunoreaction for NUCKS and low for Ki67 in IDC, grade II. (C), (F) IDC, grade III negative staining for NUCKS and strong staining for Ki67. Scale bar, 50 μm.

All the fibroadenoma cases (13/13) as well as the normal tissues that were examined, stained negatively for NUCKS (Table 1, Figures 1A, 1B). In the benign proliferative lesions examined, NUCKS was expressed in 17/23 (85%) cases of adenosis and 10/11 (91%) cases of sclerosing adenosis (Table 1, Figure 1C) exhibiting moderate staining. In the intraductal proliferative lesions, NUCKS was expressed in 13/16 (81%) cases of usual ductal hyperplasia (UDH), in 6/6 (100%) cases of atypical ductal hyperplasia (ADH) and in 15/16 (94%) cases of DCIS, exhibiting intense staining in the majority of ADH and DCIS (83% and 82% of the cases respectively stained with a score of +2/+3) (Table 1, Figures 1D, 2A).

**Table 1:** Analysis of NUCKS immunostaining in non malignant, non metastatic breast lesions

	**Fibroepithelial** **tumours** **(n=13)**	**Benign epithelial proliferations** **(n = 31)**		**Intraductal proliferative lesions** **(n = 38)**		
**Overall NUCKS** **Immuno-** **Reactivity**	**Fibroadenoma ** **(n = 13) ** **RR = ns**	**Adenosis ** **(n = 20) ** **RR = ns**	**Sclerosing adenosis ** **(n = 11)** **R = ns**	**Usual ductal hyperplasia, UDH** **(n = 16), RR = 1.5**	**Atypical ductal hyperplasia, ADH,** **(n = 6), RR = 4.0–5.0**	**Ductal carcinomas *in situ*, DCIS ** **(n = 16), RR = 8.0–11.0**

**Negative (0)**	13(100%)	3 (15%)	1 (9%)	3 (19%)		1 (6%)

**+1**		10 (50%)	6(55%)	8 (50%)	1 (17%)	2 (12%)

**+2**		6 (30%)	4(36%)	4 (25%)	2 (33%)	6 (38%)

**+3**		1 (5%)		1 (6%)	3 (50%)	7 (44%)

**Low expression** **(0/+1)**	**13 (100%)**	**13 (65%)**	**7 (64%)**	**11 (69%)**	**1 (17%)**	**3 (18%)**

**High expression (+2/+3)**		**7 (35%)**	**4 (36%)**	**5 (31%)**	**5 (83%)**	**13 (82%)**

RR = relative risk for subsequent development of invasive breast cancer; ns = non significant

Concerning the benign breast diseases examined, some of them are more closely linked to breast cancer risk than others. According to World Health Organization (WHO) [15], adenosis and sclerosing adenosis are not associated with increased risk of subsequent carcinoma. In addition, UDH is associated with a slightly elevated risk, although not considered a precursor lesion. On the other hand, ADH is associated with a moderately increased relative risk (RR 4.0–5.0) while DCIS is considered a precursor lesion (obligate or non-obligate) with a RR of 8.0–11.0 for the development of breast cancer. The results showed that NUCKS is highly expressed in ADH and DCIS, characterized of higher RR (approximately 83% of cases stained with a score of +2/+3), compared to UDH and adenosis/sclerosing adenosis, characterized of low RR (approximately 35% of cases stained with a score of +2/+3).

In breast carcinomas, the majority of the cases were stained positively for NUCKS. In grade I carcinomas, 6/9 (66.5%) cases stained positively whereas in grade II, 37/41 (90%) cases stained positively for NUCKS (Table 2, Figure 2B). In IDC, grade III, although most of the cases stained positively for NUCKS (6/8, 75%), the observed immunoreaction was low (7/8, 87.5%) (Table 2, Figure 2C).

**Table 2:** Analysis of NUCKS immunostaining in invasive breast carcinomas

**Overall NUCKS immuno-** **Reactivity**	**IDC, ILC, grade I ** (n = 9)*	**IDC, ILC, grade II ** (n = 41)**	**IDC, grade III ** **(n = 8)**
**Negative (0)**	3 (33.5%)	4 (10%)	2 (25%)

**+1**	3 (33.5%)	9 (22%)	5 (62,5%)

**+2**	2 (22%)	12 (29%)	1 (12,5%)

**+3**	1 (11%)	16 (39%)	0

**Low expression (0/+1)**	6 (67%)	13 (32%)	7 (87,5%)

**High expression (+2/+3)**	3 (33%)	28 (68%)	1 (12,5%)

* The 9 cases of grade I consisted of 8 IDC and 1 ILC

* The 41 cases of grade II consisted of 35 IDC and 6 ILC

Among the breast carcinoma cases examined, there was a relatively small number of lobular carcinomas, which in their majority exhibited low or negative staining for NUCKS. More specifically, there was one case of ILC, grade I, which was negative for NUCKS staining and 6 cases of ILC, grade II, of which 2/6 (33%) stained negatively, 2/6 (33%) low and 2/6 (33%) moderately.

Statistical analysis of all data showed no significant association between NUCKS expression and the ER, PR and HER-2/neu status of the tumors (p = 0.45; p = 0.51; p = 0.74 respectively). Furthermore, NUCKS expression was not associated with lymph node metastasis (p = 0.49).

### Primary cultures

**Figure 3:**
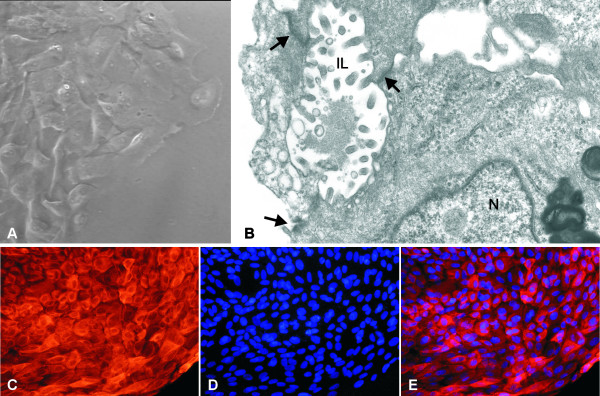
**The epithelial origin of the primary culture cells**. (A) Phase contrast micrograph showing epithelial cells derived form the tissue fragment of the breast cancer biopsy. The cells are polygonal and have pleomorphic nuclei with prominent nucleoli. (B) Electron micrograph showing the contact of adjacent epithelial breast neoplastic cells through the formation of tight junctions and desmosomes (arrows) which delineate intercellular lumina (IL) lined by protruding microvilli. N; nucleus. (C) immunofluorescence micrograph of cells from primary culture showing cytoplasmic localization of CK8 with varying intensity. (D) Cells were counterstained with DAPI. (E) Merged immunofluorescence micrographs of cytokeratin 8 localization and DAPI revealing the cytoplasmic localization of CK8 in the entire cell population verifying their epithelial origin. Original magnification: (a) ×400×, (b) ×15,400, (c-e) ×200.

Using the explant technique, the majority of cell population consists of epithelial cells since the majority of stromal cells do not migrate out of the tissue. Supplementary, morphometric analysis of each culture, revealed a small percentage of fibroblasts (<5–8%) in the entire cell population. The morphological examination of the primary culture cells with the inverted microscope, under phase-contrast field, demonstrated a typical neoplastic epithelial morphology for the majority of the tumor-derived cells (Figure 3A). They were characterized of polygonal shape with large pleomorphic nuclei and prominent nucleoli. At the ultrastructural level (Figure 3B), the epithelial neoplastic cells were attached to each other with tight junctions and desmosomes. In addition, intercellular lumina were often formed with protruding microvilli into the lumen. The epithelial origin of the cells was also verified by the CK8 immunofluorescence applied on the primary culture cells. The results showed CK8 immunofluorescence of varied intensity in the cytoplasm of the entire cell population (Figure 3C).

### Semi-quantitative NUCKS mRNA expression in primary culture cells

**Figure 4:**
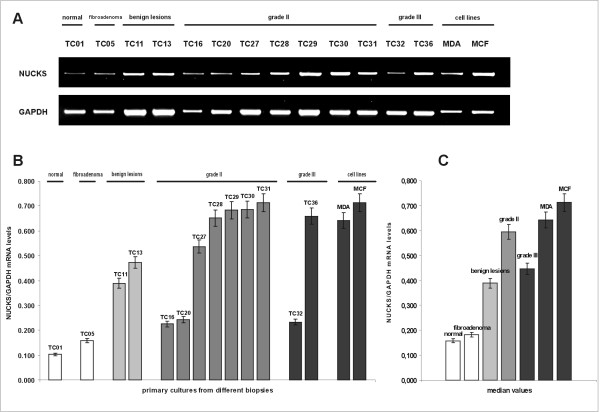
**Semi-quantitative mRNA expression of *NUCKS *in primary cultures from different biopsies**. (A) The RT-PCR products, generated with *NUCKS *and *GAPDH *gene specific primers, were electrophorized in a 2% agarose gel. *GAPDH *mRNA was used as an internal control. The most representative cases are illustrated. (B) Graphical presentation of the ratio of *NUCKS *to *GAPDH *mRNA levels corresponding to the samples illustrated in (A), as median values of 3 independent experiments (p = 0.05). The mRNA levels were quantitated semiquantitatively as described in the Methods section. TC01, primary culture of normal tissue; TC05, primary culture of fibroadenoma; TC11 and TC13, derived from primary cultures from biopsies with benign epithelial proliferations; TC16, TC20, TC27-TC31 derived from primary cultures from IDC, grade II biopsies; TC32 and TC36 derived from IDC, grade III. The clinicopathological variables of the samples are summarized in Additional file 1. MDA MB-231 and MCF-7 represent cell lines used as a positive control for NUCKS expression. (C) Median values of the ratio of NUCKS to GAPDH mRNA levels in the studied groups (p = 0.05).

Using semi-quantitative RT-PCR, the mRNA levels of *NUCKS *were evaluated in primary culture cells. The clinicopathological parameters of breast biopsies used for primary cultures are summarized in Additional file 1. In Figure 4, the representative results of each clinicopathological category are demonstrated. Specifically, in the primary cultures from normal tissues and fibroadenomas, NUCKS mRNA was detected at low levels. In the cultures derived from tissues with benign lesions, NUCKS mRNA varied from low levels, as observed in normal tissues and fibroadenomas, to higher levels resembling some cases of IDC, grade II. In most of the cultures derived from grade II tumors, NUCKS mRNA was significantly increased compared to normal. However, there were some cultures from grade II carcinomas exhibiting low NUCKS mRNA levels. The observed variation of NUCKS mRNA in primary cultures from benign and grade II biopsies is concomitant to immunohistochemical results from the corresponding biopsies (Tables 1 and 2) and may be related to tumor heterogeneity. In most of the cultures derived from biopsies of IDC, grade III, NUCKS mRNA levels were lower than that observed in primary cultures from IDC, grade II, while in few cases the levels of NUCKS mRNA expression were significantly higher.

In general, the NUCKS mRNA levels in primary cultures resembled the expression pattern in the corresponding biopsies, verifying that primary cultures retain most of the aspects of the primary tumors of origin.

### Quantitation of NUCKS mRNA levels

**Figure 5:**
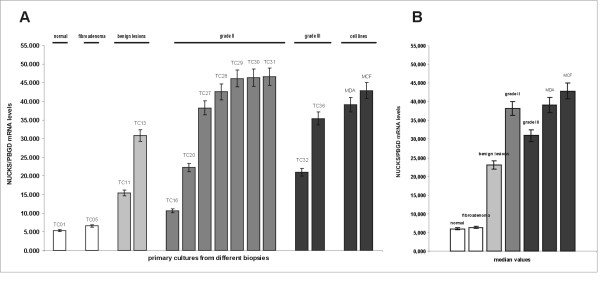
**Quantitative mRNA expression of *NUCKS *in primary cultures from different biopsies**. (A) Graphical presentation of the ratio of *NUCKS *to *PBGD *mRNA expression quantitated by qRT PCR as median values of 3 independent experiments (p = 0.05) per culture. The most representative cases are illustrated. TC01, primary culture of normal tissue; TC05, primary culture of fibroadenoma; TC11 and TC13, derived from primary cultures from biopsies with benign epithelial proliferations; TC16, TC20, TC27-TC31 derived from primary cultures from grade II breast cancer biopsies; TC32 and TC36 derived from IDC, grade III. The clinicopathological variables of the samples are summarized in Additional file 1. MDA MB-231 and MCF-7 represent cell lines used as a positive control for NUCKS expression. (B) Median values of the ratio of *NUCKS *to *PBGD *mRNA expression in the studied groups (p = 0.05).

Quantitative, real-time RT-PCR analysis of primary culture cells derived from benign lesions and breast carcinomas revealed higher NUCKS gene expression compared to normal and fibroadenoma culture cells (Figure 5). The median relative NUCKS mRNA/PBGD mRNA ratio was 5.346 (range, 3.821–6.897) for normal and 6.328 (range, 5.727–7.583) for fibroadenoma samples. Significantly higher ratio of NUCKS mRNA/PBGD mRNA was observed in benign lesions [median relative ratio 23.114 (range, 5.942–32.241)], as well as in grade II samples [median relative NUCKS mRNA/PBGD mRNA ratio 38.212 (range, 10.594–46.604)], whereas the median relative NUCKS mRNA/PBGD mRNA ratio of grade III samples was 30.962 (range, 20.929–35.390).

### Western immunoblot analysis

**Figure 6:**
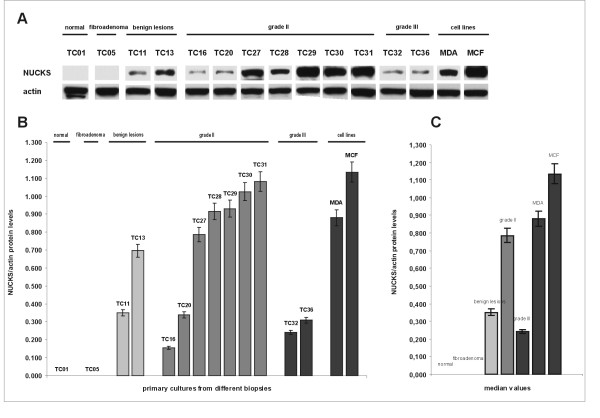
**Western immunoblot analysis of NUCKS in primary cultures from different biopsies**. (A) Equal amounts of total protein (30 μg) were subjected to immunoblot analysis with anti-NUCKS antibody. After stripping, the membrane was reprobed with anti-actin antibody. Actin protein was used as an internal control and in order to verify equal loading of the gel. MDA MB-231 and MCF-7 cell lines were used as a positive control. The most representative cases are illustrated. (B) Graphical presentation of the ratio of NUCKS to actin protein levels corresponding to the samples illustrated in (A), as median values of 2 independent experiments (p = 0.05). The protein levels were calculated from the immunoblot reaction with the Image-Pro Plus software, as described in the Methods section. TC01, primary culture of normal tissue; TC05, primary culture of fibroadenoma; TC11 and TC13, derived from primary cultures from biopsies with benign epithelial proliferations; TC16, TC20, TC27-TC31 derived from primary cultures from grade II breast cancer biopsies; TC32 and TC36 derived from IDC, grade III. NUCKS protein levels resemble its mRNA expression, exhibiting the same variation among the different samples except for IDC grade III. MDA MB-231 and MCF-7 represent cell lines used as a positive control for NUCKS expression. The clinicopathological variables of the samples are summarized in Additional file 1. (C) Median values of the ratio of NUCKS to actin protein levels in the studied groups (p = 0.05).

As shown in Figure 6, the levels of the NUCKS protein in primary cultures demonstrated a similar expression pattern compared to immunohistochemistry (Figures 1, 2), as well as to RT-PCR (Figure 4) and qRT-PCR (Figure 5). Specifically, in the primary cultures from normal tissues and fibroadenomas, the NUCKS protein levels were non detectable. In primary cultures from benign lesions, NUCKS protein levels demonstrated a variation from non detectable to higher levels. The majority of cultures derived from grade II tumors showed increased levels of NUCKS protein compared to few ones exhibiting low to non detectable protein levels. However, in the primary cultures derived from IDC, grade III biopsies the NUCKS protein levels were lower than most of grade II and benign proliferation samples.

The correlation between NUCKS mRNA and protein levels in each examined sample showed limited variation, except for grade III biopsies. Specifically, there was a direct correlation between mRNA and protein levels in normal, fibroadenoma, proliferative lesions and grade II primary cancer samples in contrast to grade III samples where NUCKS mRNA levels were significantly higher than protein levels. Actually, this finding was in full agreement with the immunohistochemical results from the corresponding IDC, grade III biopsies which stained for NUCKS with a score of +1, compared to most of the benign proliferations and grade II biopsies which stained with a score of +2 to +3. All primary cultures from benign lesions derived from biopsies with UDH along with adenosis and/or sclerosing adenosis. In these cultures there was direct correlation between mRNA levels (as evaluated with RT-PCR and qRT-PCR) and protein levels (as evaluated with western immunoblot and immunohistochemistry).

### Correlation between NUCKS and Ki67 expression

Since NUCKS is believed to be important in cell cycle progression, in order to verify that the observed NUCKS overexpression in non malignant and malignant lesions was not a result of enhanced proliferation, Ki67 expression was studied by means of immunohistochemistry and immunofluorescence in tissue sections and primary cultures respectively.

**Figure 7:**
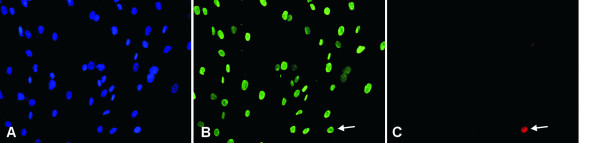
**Immunofluorescence expression of NUCKS and Ki67 in a representative primary culture derived from IDC, grade II**. The scarce expression of Ki67 and the ubiquitous expression of NUCKS are demonstrated. (A), cells counterstained with DAPI. (B), NUCKS expression in all of the cell nuclei. (C), only one cell in the optical field expressing Ki67 (arrow). Original magnification ×200.

In the tissue sections from benign epithelial proliferations, where NUCKS is found to be overexpressed, Ki67 expression was low with less than 10% of the cell nuclei staining positively (Figures 1E–1H). On the contrary, in some cases of IDC, grade II and III, there was an increased expression of Ki67 (>10%) whereas NUCKS expression was low (Figure 2C, 2F). However, in some cases of IDC, grade II, there was an increased expression of both NUCKS (score +2, +3) and Ki67 (>10%). Statistical analysis revealed no apparent correlation between NUCKS and Ki67 expression (p = 0.10). Moreover, in all of the primary cultures developed, Ki67 was scarcely expressed (Figure 7C). On the contrary, all of the cells in the primary culture exhibited variable but detectable levels of NUCKS (Figure 7B) indicating that NUCKS expression is not necessarily related with cell proliferation in breast cancer.

## Discussion

The results show that there is an increased NUCKS expression in breast cancer compared to normal tissue. More specifically, the immunohistochemical results show that the percentage of NUCKS positive cases in invasive breast cancer is increased in grade I (6/9, 67%), grade II (37/41, 90%) and III (6/8, 75%) carcinomas compared to normal cases. NUCKS is also overexpressed in many of the benign epithelial proliferations and intraductal lesions examined in this study. It is worth mentioning that the cases of ADH and DCIS showed intense staining of NUCKS. On the contrary, all the fibroadenomas and the majority of the lobular carcinomas stained negatively for NUCKS. These results are confirmed by RT-PCR, qRT-PCR and Western immunoblot analyses from the primary cultures.

The *NUCKS *gene is located on chromosome 1q32.1. There are several scientific reports utilizing array-based Comparative Genomic Hybridization (CGH) techniques, which show that there is a high frequency (59–74%) of gain of chromosome region 1q.32 in breast cancer and this gain is an early event in the cancer progression [16-21]. Most of the reports show that lobular carcinomas exhibit a higher frequency of gain of chromosomal region 1q.32 (74%) compared to ductal (59%), having however a lower total number of genetic aberrations compared to ductal carcinomas [16-21]. Furthermore, a number of genetic aberrations have been described in fibroadenomas, but none of them include the gain of chromosomal region 1q32 [22]. These data could explain the upregulation of NUCKS in ductal breast cancer as validated from the present study, and also the finding that all the fibroadenomas evaluated stained negatively for NUCKS. However, most of the lobular carcinomas stained negatively for NUCKS. It could be hypothesized that gain of chromosomal region 1q32 is a prerequisite but not sufficient for the increase of NUCKS expression, and that an additional background of genetic aberrations and instability is responsible for triggering the upregulation of NUCKS mRNA and protein.

Noteworthy, there was a correlation between NUCKS overexpression and grading of DCIS. Low grade, well differentiated DCIS exhibited intense staining for NUCKS like the majority of ADH lesions. On the contrary, high grade poor differentiated DCIS, like most of the IDC, grade III cases, exhibited weak to moderate NUCKS staining. This finding is in accordance with the view of Simpson et al [23] that ADH and low grade DCIS show highly similar pathological and genetic features and constitute a distinct entity, whereas high grade DCIS and grade III carcinomas constitute a different pathological and genetic entity.

NUCKS has been characterized as a cell cycle related protein synthesized during the M/G1 phase [7]. However, the fact that its expression is not correlated with Ki67 proliferating index, neither in breast tissue biopsies nor in primary culture cells, as demonstrated in the present study, implies that NUCKS has a more complex role than just promoting cell proliferation. Moreover, there are a number of scientific data indicating that NUCKS could play a role in the DNA damage response. Although NUCKS sequence shares no homology with any other protein in protein databases or translated cDNA databases [24], it exhibits a slight homology with RAD51AP1, a nucleic acid binding protein interacting with Rad51 recombinase in maintaining genomic integrity and possibly implicated in DNA damage response [25]. Furthermore, NUCKS is a substrate for ATM (ataxia telangiectasia mutated) and ATR (ATM and Rad3-related) kinases after irradiation of 293T cells [26], implying that NUCKS is a cell cycle related protein involved in DNA damage response. Since the activation of the DNA damage checkpoint is an early event in carcinogenesis [27], it could be proposed that the observed NUCKS overexpression in benign epithelial proliferations and intraductal lesions is related with the activation of DNA damage checkpoints.

Gene expression profiling of breast tumors is a novel tool in the effort to classify tumor subtypes, predict risk of relapse, identify genes that mediate disease progression and select optimal therapeutic options [28]. Among the gene signatures established so far concerning breast cancer (for a complete review see [29]), *NUCKS *has been found to correlate with the invasive gene signature (IGS) [30]. The IGS consists of 186 genes which are overexpressed in breast cancer stem cells identified by high expression of CD44 and low expression of CD24 (CD44+CD24-/low), compared to normal breast epithelial cells. The CD44+CD24-/low cells represent a small subclass within the tumor but are considered to be the actual tumorigenic cells whereas the rest, called nontumorigenic, have little or no such ability. The fact that *NUCKS *belongs to IGS demonstrates its important role in breast cancer but further investigation is needed in order to elucidate whether the observed overexpression in breast cancer is correlated with oncogenic progression and poor prognosis or reflect a preventive response to cancer-related genomic instability.

There is still much work to be done on elucidating the mechanisms by which NUCKS contributes to oncogenesis so that in the long term this protein could serve as a potential prognostic marker for breast cancer progression.

## Conclusion

For the first time, an evaluation of NUCKS protein levels is made in association with the histopathological types and grade by means of immunohistochemical, biochemical and molecular techniques applied on benign, intraductal and cancerous breast lesions. The results show overexpression of the NUCKS protein in a number of non malignant breast lesions and cancerous tissues. In particular, the NUCKS overexpression in ADH and DCIS indicates a future significant role of this protein in evaluating neoplastic progression.

## Methods

### Archival material

The material of this study was obtained from Evgenidion University Hospital and consisted of 91 paraffin blocks from female patients who underwent diagnostic biopsy or radical mastectomy from 2004 onwards. All the breast tissues biopsies were histological classified according to current World Health Organization (WHO) scale [15]. The tissue biopsies included 20 cases of benign epithelial proliferations (adenosis and sclerosing adenosis), the majority of which coexisted with usual ductal hyperplasia (UDH); 13 cases of fibroadenomas; 8 cases of IDC, grade I; 35 cases of IDC, grade II; 8 cases of IDC, grade III; 1 case of ILC, grade I and 6 cases of ILC, grade II. Sixteen ductal carcinoma in situ cases (DCIS) co-existing with IDC lesions of different histological grading were also studied. Specifically, 2 cases of DCIS were co-existed with IDC, grade I; 12 cases of DCIS were co-existed with IDC, grade II and 2 cases of DCIS were co-existed with IDC, grade III. Furthermore, among the studied material, 6 cases of atypical ductal hyperplasia (ADH) were encountered as components of some of the above lesions. Particularly, 1 case of ADH co-existed with benign lesions; 1 case with IDC, grade I; 3 cases with IDC, grade II and 1 case with IDC, grade III. The histological grading of in-situ carcinomas was evaluated according to Holland et al [31]. The histological grading of invasive breast carcinomas was evaluated according to the modified Scarff-Bloom-Richardson histological grading system with guidelines as suggested by Nottingham City Hospital pathologists [32].

The normal breast tissues refer to areas of paraffin sections next to benign epithelial proliferations. Information on axillary lymph node status was available in all cases. Biomarkers concerning ER, PR and HER-2/neu were provided from the Pathology Department of Evgenidion Hospital. All investigational biopsies were approved by the Athens Medical School Ethical Committee (approval no.5758/12-3-03).

### Primary cell cultures

For primary cell culturing, 33 breast tissue biopsies were obtained and processed-in parallel to the paraffin embedding procedure- as previously described [33]. In addition, 3 biopsies of normal tissue derived from areas adjacent to benign epithelial proliferations were also processed for primary culturing. From each biopsy, several small (2–3 mm^3^) tissue fragments were placed onto plastic Petri dishes 35 mm in diameter and cultured in RPMI 1640 supplemented with 10% Fetal Calf Serum (FCS), 1 mM L-glutamine, 1% penicillin and 1% streptomycin, at 37°C, under 5% CO_2_. The cells were allowed to migrate out of the tissue fragment using the explant technique, without any mechanical or enzymatic tissue disaggregation. The cell growth was complete in about 2–3 weeks. The tissue biopsies were marked as TC01-TC36 [see Additional file 1].

Cell culturing and further processing of the tissue were carried out at the Laboratory of Histology and Embryology, Medical School, University of Athens.

### Cell line cultures

The human breast cancer cell lines MDA-MB-231 and MCF-7 were used as positive controls for NUCKS expression. MCF-7 cell line was also used as a positive control for cytokeratin 8 (CK8) and Ki67 expression using indirect immunofluorescence. The cells were grown in RPMI 1640 medium supplemented with 10% Foetal Calf Serum (FCS), 1 mM L-glutamine, 1% penicillin, and 1% streptomycin. Cells were incubated at 37°C, under 5% CO_2_.

### Transmission electron microscopy

For the ultrastructural confirmation of the epithelial origin, cells of primary cultures were fixed in 2.5% glutaraldehyde made up in phosphate buffer saline (PBS) 0.01 M, pH 7.4, for 15 min at room temperature, postfixed in 1% aqueous OsO4 for 30 min at 4°C, isolated according to Kouloukoussa et al. [34] and were finally embedded in a mixture of epoxy resins. Ultrathin (80–90 nm thick) sections were cut with a Diatome diamond knife on a Leica Ultracut R ultramicrotome and were collected onto 200 mesh uncoated copper grids.

### Paraffin immunohistochemistry

The preparation of the antibody against NUCKS protein has been described in details in Østvold et al, 2001 [4] and it has been used in several publications [11,24]. In the present study, the specificity of the Ab was first checked in immunoblot with protein extracts from MCF-7 cells.

Tissue sections (5 μm thick) were obtained from representative formalin-fixed paraffin-embedded tissue blocks from each patient. The sections were mounted on Superfrost® slides and deparaffinized by heating at 60°C for 30 min followed by immersion in xylene for 2 × 10 min and rehydrated by immersion in 100% ethanol for 2 × 5 min, 95% ethanol for 5 min, 70% ethanol for 5 min, 50% ethanol for 5 min and dH_2_O for 2 × 5 min. In order to inactivate endogenous peroxidase activity, the slides were incubated in 3% H_2_O_2 _for 30 min followed by immersion in dH_2_O 3 × 5 min. Antigen retrieval was performed by trypsin (0,1% trypsin in 0,1% CaCl, 20 mM Tris-Cl, pH 7,8) digestion for 10 min at 37°C. The slides were rinsed with tap water for 5 min followed by immersion in dH_2_O for 3 × 5 min and in PBS for 3 × 5 min. The slides were then incubated with blocking solution PBS, 1% BSA, 5% NGS for 1 h at RT followed by incubation with primary affinity purified rabbit anti-NUCKS Ab (1:300) in PBS, 1% BSA, 5% NGS overnight at 4°C. The next day, the slides were rinsed in PBS for 3 × 5 min and incubated with HRP conjugated secondary goat anti-rabbit Ab (1:100) in PBS, 1% BSA for 1 h at RT, followed by immersion in PBS for 3 × 5 min. The signal was visualized with DAB chromogen (0.05% DAB, 0.024% H_2_O_2 _in PBS). The slides were then stained with hematoxylin, dehydrated in alchohol, immersed in xylene and finally coverslipped. As negative controls, paraffin sections were treated with the same immunohistochemical protocol, but instead of primary Ab, were incubated with PBS, 5% NGS, 1% BSA. As positive controls, paraffin sections of whole rat embryo E13,5 p.c were used [4]. The sections were photographed under a LEICA DM LB2 photomicroscope, equipped with a DFC 320 digital camera.

Immunoreactivity was evaluated in 10 high power fields as follows: A score of 0 was given if fewer than 10% of positive nuclei of epithelial cells were detected; score 1 if 11% to 30% were detected with low staining intensity; score 2 if more than 30% and less than 60% of positive nuclei were detected with moderate staining intensity and score 3 if more than 60% of positive nuclei were detected with high staining intensity.

### Immunofluorescence of primary culture cells

Primary culture cells grown on sterilized glass coverslips were fixed for 20 min in 4% paraformaldehyde, washed in PBS and permeabilized for 5 min in 0.2% Triton X-100 in PBS. The cells were washed 3 times over 5 min in PBS and incubated with affinity purified rabbit anti-NUCKS antibody (1:600) and mouse anti-Ki67 antibody (clone: MIB-1, M7240, DAKO) (1:400), or mouse anti-cytokeratin 8 (1:100) (C5301, Sigma) for 1 h at room temperature. The cells were then washed 3 times over 5 min with PBS and incubated for 30 min at room temperature, with a mixture of Alexa-488 conjugated donkey anti-rabbit IgG antibody (1:150) and CF™555-conjugated donkey anti-mouse IgG antibody (1:200). DAPI was used as a counterstain. Stained slides were examined in a Nikon Eclipse TE 2000-S fluorescent microscope.

### RNA isolation-cDNA synthesis

Total cellular RNA was extracted from primary culture cells and cell lines by TRizol reagent according to the manufacturer's instructions (Invitrogen) and *in vitro *transcribed to cDNA using Moloney Murine Leukaemia Virus reverse transcriptase (RT) and random hexamers as primers. As a control for the presence of amplifiable cDNA, retinoic acid receptor alpha sequences (*RARα*) were amplified in separate reactions as previously described [35]. Both in the RT reaction and in the ensuing amplification reactions, recommended measures to prevent cross-contamination of samples were followed. In addition, for each experiment, a control with no template was used to check for the presence of any contaminant.

### PCR amplification of NUCKS cDNA sequences

Ten microliters of the RT reaction was amplified by PCR using oligonucleotides specific for the NUCKS gene. PCR was carried out in a final volume of 100 μl, with 50 pmol of each oligonucleotide primer, 200 μmol/l each of dNTP, 1 U of Go Taq Flexi polymerase (Promega) in PCR buffer (20 mM Tris-HCl, pH 8.4, 50 mM KCl, 1.5–2.0 mM MgCl_2_). The amplification condition were 94°C for 3 min, 30 cycles of 94°C for 30 sec, 60°C for 30 sec, 72°C for 1 min and finally 72°C for 10 min. The sequences of the PCR primers used for amplification of NUCKS cDNA are shown in Table 3.

**Table 3:** Primers and probes for semi-quantitative and real-time PCR

**Oligonucleotide**	**Sequence**
NUCKS F (forward)	5'-AATTCACAGGAAGATAGTGAGG-3'

NUCKS R (reverse)	5'-TCACTGTAG CCTTTAGTCTGGG-3'

GAPDH forward	5'-ATGGGGAAGGTGAAGGTCGGAGTC-3'

GAPDH reverse	5'-GAACATGGGGGCGTCAGCAGAG-3'

NUCKS Fq (forward, real-time PCR)	5'-TCTGATGATGCAGATGAAGATTA-3'

NUCKS Rq (reverse, real-time PCR)	5'-CTGCTGAGTGAGAATCATCC-3'

NUCKS FL probe	5'-TCTTGGTCTTCACATCTTTGTCTTCT-FL-3'

NUCKS LC probe	5'-LC640-AGTCCTCACTATCTTCCTGTGAATTCTTT-3'

Determination of the specific PCR products was also performed using direct sequencing. For the sequencing analysis, PCR specific bands were isolated and purified from 3% low melting point agarose gel and sequenced by the dideoxy-chain termination method using the modified version of T7 DNA polymerase (Sequenase 2.0, US Biochemicals, Cleveland, Ohio, USA).

### Semi-quantitative RT-PCR

Amplification of a product corresponding to a region from exon 3 to exon 7 of the *NUCKS *cDNA sequence was performed as described above. In parallel, a *GAPDH *mRNA transcript was amplified from the same samples (nucleotide sequences of the primers are shown in Table 3). The amplified products were electrophorised in a 2% agarose gel, stained with ethidium bromide, photographed under UV illumination and the images were digitally captured. All experiments were performed in triplicates.

The images were analyzed using the Image-Pro Plus, version 3.0, software for Windows (Media Cybernetics, Silver Spring, MD, USA) and the OD for *NUCKS *and *GAPDH *PCR products was calculated. Each sample was tested at least twice. The amount of cDNA corresponding to the *NUCKS *gene in each sample was determined by identification of the ratio of the intensity of the band corresponding to each sample to that of *GAPDH*.

### Quantitative, real-time PCR (qRT-PCR) assessment of NUCKS mRNA expression

In order to quantitate *NUCKS *mRNA transcripts, real-time fluorescence PCR assays were developed and evaluated for the Roche LightCycler (LC; Roche Diagnostics). After adding 180 μL water to the reverse transcription reaction product, 2 μL aliquots were used for each reaction. In a separate PCR reaction, the same cDNA was evaluated for expression of the porphobilinogen deaminase (*PBGD*) gene using the LightCycler(r) h-PBGD Housekeeping Gene Set (Roche). The amplification mixture consisted of 2 μL of 10× reaction mix (LC-FastStart master hybridization probes; Roche Diagnostics), 2 mM MgCl_2_, 0.5 μM concentration of each oligonucleotide primer, 0.15 μM concentration of each oligonucleotide probe, and 2 μL of template cDNA in a final volume of 20 μL. Samples were amplified as follows: an initial denaturation step at 95°C for 3 min to activate the FastStart Taq DNA polymerase, 40 cycles of denaturation at 95°C for 10 sec, annealing at 51°C for 15 sec and extension at 72°C for 15 sec. The temperature transition rate was 20°C/sec. All experiments were performed in triplicates.

DNA oligonucleotide primers and hybridization probes were synthesized by TIB Molbiol (Berlin, Germany). The adjacent ends of the hybridization probes were labelled with fluorophores. The 5' end of the first probe was labelled with the acceptor fluorophore LC Red 640 and the 3' end of the second probe with the donor fluorescein (FITC, 3FL). The 5' labelled probes were 3'- phosphorylated to block polymerase extension during PCR. Nucleotide sequences of the primers and hybridization probes are shown in Table 3.

### Western immunoblot analysis

Cell harvesting, sample processing, electrophoresis and immunoblot were performed as previously described [33]. In brief, 30 μg of total protein were loaded from each sample and resolved on 15% polyacrylamide SDS gel. After blocking, the membranes were incubated overnight at 4°C with rabbit anti- human NUCKS (1:2,000) antibody. The reaction was visualized with ECL Plus (Amersham) on Kodak Biomax XAR films. In order to verify equal loading, the membranes were stripped and re-probed with mouse anti-human actin (1:3,000) (MAB1501, Chemicon). All experiments were performed in duplicates. The films were scanned and densitometric measurements for NUCKS and actin bands were performed as for RT-PCR.

### Statistical analysis

The chi-square test was used to analyze the correlation between NUCKS expression by immunohistochemistry and clinicopathological features. P values of less than 0.05 were considered significant.

## Abbreviations

ER: estrogen receptors; PR: progesterone receptors; HER2/neu: human epidermal growth factor receptor-2; DCIS: ductal carcinoma *in situ*; LCIS: lobular carcinoma *in situ*; IDC: invasive ductal carcinoma; ILC: invasive lobular carcinoma; UDH: usual ductal hyperplasia; ADH: atypical ductal hyperplasia; RT-PCR: Reverse Transcription Polymerase Chain Reaction; qRT-PCR: quantitative real-time PCR; FCS: foetal calf serum; PBS: phosphate buffer saline; BSA: bovine serum albumin; HRP: horseradish peroxidise; CK8: cytokeratin 8.

## Competing interests

The authors declare that they have no competing interests.

## Authors' contributions

YD is the principal investigator. He executed all the lab work; cell culturing, RNA and protein isolation, immunohistochemistry, microscopy, photography, Western immunoblotting, RT PCR and qRT PCR. MK is a cell culture specialist who coordinated primary culture establishment, growth and maintenance. ACO is a senior scientist who participated in the study design. KG made the anti-NUCKS antibodies. NG is a pathologist responsible for tissue biopsy delivery and clinicopathological determination and for scoring the NUCKS immunoreaction. DV is a pathologist responsible for handling tissue biopsies and independently scoring the NUCKS immunoreaction. SH worked on electron microscopy, fluorescent experiments and photography. PK designed the real time PCR experiments. CK participated in experimental design and helped to draft the manuscript. EM worked as a senior microscopy specialist for the examination and photography of the samples under the fluorescent, phase contrast and electron microscope. VAM coordinated the research group as a biochemistry laboratory supervisor. All authors read and approved the final manuscript.

## Supplementary Material

Additional file 1 **histopathological characteristics**. The table includes the histological parameters of the biopsies used in the study, the expression profile of estrogen receptors, progesterone receptors, Her2/neu and the number of infiltrated lymph nodes. Click here for file
