# Supplementary material for: NUCKS overexpression in breast cancer
Source: Cancer Cell Int. 2009 Aug 10;9:19. doi: 10.1186/1475-2867-9-19 (PMC2743642; doi:10.1186/1475-2867-9-19)
Supplement: Additional file 1 — histopathological characteristics. The table includes the histological parameters of the biopsies used in the study, the expression profile of estrogen receptors, progesterone receptors, Her2/neu and the number of infiltrated lymph nodes. [file 1475-2867-9-19-S1.doc]

**Additional Table -** Clinicopathological parameters of breast biopsies used for Immunohistochemistry and primary cultures

| **Biopsy number** | **culture**  **ID** | **Tumor grade** | **Histological features** | **ER**  **Status** | **PR**  **status** | **HER2/neu**  **Status** | **Lymph node**  **Metastasis**** |
| --- | --- | --- | --- | --- | --- | --- | --- |
| **1** | **TC01** | - | Tissue area next to fibrocystic lesions | - | - | - | - |
| **2** | **TC02** | - | Tissue area next to fibrocystic lesions | - | - | - | - |
| **3** | **TC03** | - | Tissue area next to fibrocystic lesions | - | - | - | - |
| **Fibroepithelial tumors** | | | | | | | |
| **4** | **TC04** | NM | Fibroadenoma | - | - | - | - |
| **5** | **TC05** | NM | Fibroadenoma | - | - | - | - |
| **6** | **TC06** | NM | Fibroadenoma | - | - | - | - |
| **7** | **TC07** | NM | Fibroadenoma | - | - | - | - |
| **8** |  | NM | Fibroadenoma | - | - | - | - |
| **9** |  | NM | Fibroadenoma | - | - | - | - |
| **10** |  | NM | Fibroadenoma | - | - | - | - |
| **11** |  | NM | Fibroadenoma | - | - | - | - |
| **12** |  | NM | Fibroadenoma | - | - | - | - |
| **13** |  | NM | Fibroadenoma | - | - | - | - |
| **14** |  | NM | Fibroadenoma | - | - | - | - |
| **15** |  | NM | Fibroadenoma | - | - | - | - |
| **16** |  | NM | Fibroadenoma | - | - | - | - |
| **Tissues with benign epithelial proliferations and intraductal proliferative lesions (except DCIS)** | | | | | | | |
| **17** | **TC08** | NM | Sclerosing adenosis; UDH | - | - | - | - |
| **18** | **TC09** | NM | Adenosis; UDH | - | - | - | - |
| **19** | **TC10** | NM | Adenosis; UDH | - | - | - | - |
| **20** |  | NM | Adenosis; sclerosing adenosis | - | - | - | - |
| **21** |  | NM | Adenosis; UDH | - | - | - | - |
| **22** |  | NM | Adenosis | - | - | - | - |
| **23** |  | NM | Adenosis; sclerosing adenosis; ADH | - | - | - | - |
| **24** |  | NM | Adenosis; sclerosing adenosis | - | - | - | - |
| **25** |  | NM | Sclerosing adenosis | - | - | - | - |
| **26** |  | NM | Adenosis; sclerosing adenosis; UDH | - | - | - | - |
| **27** |  | NM | Adenosis | - | - | - | - |
| **28** |  | NM | Adenosis; sclerosing adenosis; UDH | - | - | - | - |
| **29** |  | NM | Adenosis; UDH | - | - | - | - |
| **30** |  | NM | Adenosis; sclerosing adenosis | - | - | - | - |
| **31** |  | NM | Adenosis; sclerosing adenosis | - | - | - | - |
| **32** |  | NM | UDH | - | - | - | - |
| **33** |  | NM | Adenosis; UDH | - | - | - | - |
| **34** | **TC11** | NM | Adenosis; sclerosing adenosis; UDH | - | - | - | - |
| **35** | **TC12** | NM | Adenosis; UDH | - | - | - | - |
| **36** | **TC13** | NM | Adenosis; sclerosing adenosis; UDH | - | - | - | - |
| **Grade I carcinomas** | | | | | | | |
| **37** |  | I | ILC | Pos(+3) | Pos(+3) | Pos(+1) | 0/6 |
| **38** |  | I | IDC | Pos(+3) | Pos(+3) | Pos(+1) | 0/23 |
| **39** |  | I | IDC; DCIS low grade* 1 | Pos(+3) | Pos(+2) | Pos(+1) | 0/26 |
| **40** |  | I | IDC | Pos(+2) | Pos(+2) | Pos(+1) | 0/2 |
| **41** |  | I | IDC; DCIS low grade* 1 | Pos(+2) | Pos(+2) | Pos(+1) | 0/2 |
| **42** |  | I | IDC | Pos(+3) | Pos(+2) | Neg | 0/9 |
| **43** |  | I | IDC | Pos(+3) | Pos(+2) | Neg | 0/7 |
| **44** |  | I | IDC | Pos(+3) | Pos(+2) | Pos(+2) | 0/4 |
| **45** |  | I | IDC; adenosis; UDH; ADH | Pos(+3) | Pos(+1) | Pos(+2) | 0/2 |
| **Grade II carcinomas** | | | | | | | |
| **46** | **TC14** | II | IDC | Pos(+1) | Pos(+1) | Pos(+3) | 0/26 |
| **47** | **TC15** | II | IDC | Pos(+3) | Pos(+3) | Pos(+1) | 0/26 |
| **48** | **TC16** | II | IDC | Pos(+2) | Pos(+2) | Neg | 6/11 |
| **49** | **TC17** | II | IDC | Pos(+2) | Pos(+1) | Neg | 0/2 |
| **50** | **TC18** | II | IDC; DCIS high grade* 3 | Pos(+3) | Pos(+2) | Neg | 1/13 |
| **51** | **TC19** | II | IDC | Pos(+3) | Pos(+3) | Pos(+1) | 0/15 |
| **52** | **TC20** | II | ILC | Pos(+3) | Pos(+3) | Pos(+3) | 1/20 |
| **53** | **TC21** | II | IDC; DCIS intermediate grade* 2 | Pos(+3) | Pos(+1) | Pos(+2) | 16/28 |
| **54** | **TC22** | II | IDC; DCIS low* 1; ADH | Pos(+3) | Pos(+2) | Pos(+1) | 1//14 |
| **55** |  | II | ILC | Pos(+3) | Pos(+2) | Pos(+1) | 0/2 |
| **56** |  | II | ILC | Pos(+3) | Pos(+2) | Pos(+1) | 0/11 |
| **57** |  | II | IDC; DCIS low grade* 1 | Pos(+1) | Pos(+2) | Neg | 0/5 |
| **58** | **TC23** | II | IDC | Pos(+3) | Pos(+2) | Pos(+3) | 0/12 |
| **59** | **TC24** | II | IDC | Pos(+3) | Pos(+1) | 0 | 3/16 |
| **60** | **TC25** | II | IDC; DCIS intermediate grade* 2 | Pos(+2) | Pos(+2) | Pos(+2) | 3/31 |
| **61** | **TC26** | II | IDC | Pos(+3) | Pos(+2) | 0 | 0/15 |
| **62** |  | II | IDC | Pos(+3) | Pos(+3) | Pos(+2) | 0/20 |
| **63** |  | II | IDC | Pos(+3) | Pos(+2) | 0 | 2/17 |
| **64** |  | II | IDC | Pos(+3) | Pos(+2) | 0 | 2/18 |
| **65** |  | II | IDC | Pos(+3) | Pos(+3) | Pos(+3) | 4/11 |
| **66** |  | II | IDC | Pos(+3) | Pos(+2) | Pos(+2) | 2/12 |
| **67** |  | II | IDC | Pos(+3) | Pos(+2) | Pos(+1) | 1/20 |
| **68** |  | II | IDC | NA | NA | NA | 0/26 |
| **69** |  | II | IDC | Pos(+3) | Pos(+2) | Pos(+2) | 0/2 |
| **70** |  | II | IDC | Pos(+3) | Pos(+2) | Pos(+1) | 0/2 |
| **71** |  | II | ILC | Pos(+3) | Pos(+1) | Pos(+2) | 0/3 |
| **72** |  | II | IDC | Pos(+3) | Pos(+1) | Pos(+2) | 0/2 |
| **73** |  | II | IDC; DCIS intermediate grade* 2 | Pos(+3) | Neg | Pos(+1) | 0/2 |
| **74** |  | II | IDC | Pos(+3) | Pos(+3) | Pos(+2) | 1/11 |
| **75** |  | II | IDC | Pos(+2) | Pos(+2) | Pos(+1) | 0/1 |
| **76** |  | II | IDC; DCIS low grade* 1 | Pos(+3) | Neg | Pos(+3) | 0/2 |
| **77** |  | II | IDC | Neg | Neg | Pos(+3) | 2/19 |
| **78** |  | II | ILC; adenosis; UDH | Pos(+3) | Pos(+1) | Pos(+2) | 27/33 |
| **79** | **TC27** | II | IDC; DCIS high grade* 3 | Pos(+3) | Neg | Pos(+3) | 1/21 |
| **80** | **TC28** | II | IDC; DCIS low grade* 1 | Pos(+3) | Neg | Pos(+2) | 1/15 |
| **81** | **TC29** | II | IDC; DCIS intermediate grade* 2 | Pos(+2) | Neg | Pos(+1) | 0/5 |
| **82** | **TC30** | II | IDC; DCIS low grade* 1; UDH; ADH | Pos(+3) | Pos(+1) | Pos(+2) | 0/3 |
| **83** | **TC31** | II | IDC | Pos(+3) | Pos(+1) | Pos(+1) | 1/1 |
| **84** |  | II | IDC; DCIS intermediate grade* 2; sclerosing adenosis, UDH, ADH | Pos(+3) | Pos(+2) | Pos(+2) | 2/25 |
| **85** |  | II | ILC | Pos(+3) | Pos(+2) | Pos(+1) | 0/11 |
| **86** |  | II | IDC | Pos(+3) | Neg | Pos(+1) | 0/2 |
| **Grade III carcinomas** | | | | | | | |
| **87** | **TC32** | III | IDC | Pos(+1) | Pos(+1) | Neg | 3/16 |
| **88** | **TC33** | III | IDC; DCIS high grade* 3 | Pos(+2) | Pos(+2) | Pos(+2) | 0/3 |
| **89** | **TC34** | III | IDC | Pos(+2) | Neg | Pos(+3) | 7/21 |
| **90** |  | III | IDC | Neg | Neg | Pos(+2) | 7/28 |
| **91** | **TC35** | III | IDC, DCIS high grade* 3 | Pos(+3) | Pos(+2) | Pos(+3) | 0/25 |
| **92** | **TC36** | III | IDC, adenosis, ADH | Pos(+1) | Pos(+2) | Neg | 0/2 |
| **93** | **TC36** | III | IDC | Pos(+3) | Neg | Pos(+2) | 0/17 |
| **94** |  | III | IDC | Neg | Pos(+2) | Pos(+2) | 2/18 |

NM, nonmalignant; NA, not applicable; UDH, usual ductal hyperplasia; ADH, atypical ductal hyperplasia; IDC, invasive ductal carcinoma; ILC, invasive lobular carcinoma; DCIS, ductal carcinoma *in situ*; Neg, negative; Pos, positive; * the histological grading of in situ carcinomas was evaluated according to Holland et al [31]; ** number of lymph nodes found infiltrated from cancer cells out of the total number of lymph nodes examined.
